# Benefits of three consecutive sputum inductions using nebulized racemic salbutamol versus hypertonic saline in smear-negative or sputum-scarce pulmonary tuberculosis patients: a prospective randomized, double-blind study

**DOI:** 10.1128/spectrum.00581-26

**Published:** 2026-07-02

**Authors:** Warangkana Keeratichananont, Suriya Keeratichananont, Natthaphon Ubonsutvanich, Jirachaya Chaisurote

**Affiliations:** 1 Division of Respiratory and Respiratory Critical Care Medicine, Department of Internal Medicine, Faculty of Medicine, Prince of Songkla University, Songkhla, Thailand; 2 NKC Institute of Gastroenterology and Hepatology, Faculty of Medicine, Prince of Songkla University, Songkhla, Thailand; 3 Department of Internal Medicine, Faculty of Medicine, Prince of Songkla University, Songkhla, Thailand; Laboratory Corporation of America Holdings, Burlington, North Carolina, USA

**Keywords:** tuberculosis, pulmonary, induced, hypertonic, agents, sputum, saline solution, bronchodilator, adrenergic beta-2 receptor agonists

## Abstract

Although, sputum induction (SI) with hypertonic saline increases the diagnostic yield for suspected pulmonary tuberculosis (PTB) in patients with smear-negative microscopy, but, it carries adverse effects (AEs), particularly bronchospasm. This study assessed and compared the diagnostic yields and safety profiles of three consecutive SI procedures using nebulized salbutamol vs conventional 3% sodium chloride (NaCl) in patients with suspected PTB and initially negative sputum smears. A randomized, double-blind study was conducted at Songklanagarind Hospital, Thailand, between 2016 and 2019. Three hundred forty-five adults with suspected smear-negative or sputum-scarce PTB were randomized to undergo 3 consecutive days of SI with either nebulized salbutamol (*n* = 172) or 3% NaCl (*n* = 173). Diagnostic yields were assessed using acid-fast bacilli (AFB) smear and culture for *Mycobacterium tuberculosis*. Nebulized salbutamol produced a significantly higher overall diagnostic yield by AFB smear or culture than 3% NaCl (18.0% vs 9.2%, *P* = 0.043). For culture, both cumulative and incremental yields were consistently higher in the salbutamol group across all three samples (all *P* < 0.050). The 3% NaCl group experienced significantly more overall SI-related AEs than the salbutamol group (28.3% vs 9.8%, *P* = 0.034). Specifically, chest tightness (8.1% vs 2.3%, *P* = 0.031) and bronchospasm requiring rescue bronchodilators (13.3% vs 0.0%, *P* < 0.001) were more frequent in the 3% NaCl group. Three consecutive SI using nebulized salbutamol provide higher overall and cumulative diagnostic yields than conventional hypertonic saline, primarily driven by culture positivity, while offering a superior safety profile by eliminating bronchospasm risk. Nebulized salbutamol should, therefore, be considered the preferred induction agent for SI, particularly in high-risk patients such as those with airway diseases.

**IMPORTANCE:**

Delays in diagnosing pulmonary tuberculosis or initiating treatment without bacteriological confirmation increase mortality, often because of inadequate sputum samples or negative acid-fast bacilli smears from self-expectoration. Sputum induction with hypertonic saline can improve diagnostic yield in patients with scanty sputum or smear-negative disease, but it is associated with adverse effects—particularly bronchospasm—which may require stopping the procedure. We demonstrated that three consecutive sputum inductions using nebulized racemic salbutamol produced significantly higher overall diagnostic yields by acid-fast bacilli smear and *Mycobacterium tuberculosis* culture compared with 3% NaCl. For culture, both cumulative and incremental yields were consistently superior in the salbutamol group. Regarding safety, 3% NaCl caused more chest tightness and symptomatic bronchospasm. These findings highlight the diagnostic benefit and favorable safety profile of three consecutive salbutamol-induced sputum procedures, supporting its use as the preferred agent for sputum induction.

## INTRODUCTION

Pulmonary tuberculosis (PTB) remains a major global health threat. In 2023, 1.25 million (11.6%) deaths were recorded (1). Early detection and prompt initiation of effective treatment are, therefore, critical for TB control (2). For early detection, individuals clinically suspected to have active PTB, two to three sputum samples should be collected to identify *Mycobacterium tuberculosis* (MTB) using acid-fast bacilli (AFB) smear, rapid molecular tests, and culture (3). So, sputum remains the cornerstone specimen for bacteriological confirmation, assessment of infectiousness, and monitoring of treatment response (4). However, 30%–65% of PTB patients have either smear-negative (by self-expectoration) or sputum-scarce disease (5–8). These groups are strongly associated with diagnostic delays and higher mortality (9–11). Therefore, sputum induction has been recommended as a subsequent step to facilitate rapid presumptive diagnosis (3, 7, 12).

Sputum induction (SI) with 3%–7% hypertonic saline has been shown to increase the diagnostic yield for PTB, with a pooled estimate of 74%, and further improvement when the procedure is repeated (13–16). However, SI with hypertonic saline can cause adverse effects, including chest tightness, hemoptysis, oxygen desaturation, and bronchospasm, leading to the termination of the procedure in up to 23% of cases. Therefore, the development and evaluation of novel induction agents should be considered (17–22).

β₂-Agonists, such as salbutamol and fenoterol, are commonly used bronchodilators that enhance mucociliary transport and facilitate expectoration—properties that may benefit SI (23–27). In 2015, the authors conducted a randomized controlled trial (RCT) (19) comparing the efficacy of a single SI using 5 mg nebulized racemic salbutamol vs 4 mL of 3% sodium chloride (NaCl) in 112 patients with smear-negative or sputum-scarce suspected PTB. SI with salbutamol achieved diagnostic yields comparable to 3% NaCl (positive AFB smear: 15% vs 13%; MTB culture: 22% vs 17%; *P* = 0.5) but resulted in significantly fewer AEs (10.2% vs 34.0%; *P* = 0.02) and no symptomatic bronchospasm (0% vs 11.3%; *P* = 0.02).

Recently, in 2025 (28), Mingchay et al. conducted an RCT comparing one to three rounds of SI using a combination regimen—nebulized bronchodilators (fenoterol 0.62 mg and ipratropium bromide 0.25 mg) in 2 mL plus 3 mL of 3% NaCl—vs 5 mL of 3% NaCl alone in 208 patients with suspected PTB who had negative initial spot sputum Xpert MTB/RIF Ultra results or were sputum-scarce. The combination regimen resulted in significantly higher diagnostic yields when tested with Xpert MTB/RIF Ultra, as well as fewer AEs.

However, only a few RCTs have evaluated the benefits of bronchodilator-enhanced SI, and all have used participant-blind designs that may introduce bias in assessing induction effects. We, therefore, conducted a double-blind study to compare the diagnostic yield and safety of SI using nebulized racemic salbutamol vs 3% NaCl in patients with suspected active PTB who were smear-negative or sputum-scarce, employing three consecutive SI procedures.

## MATERIALS AND METHODS

A prospective, randomized, double-blind, comparative study was conducted at Songklanagarind Hospital, Songkhla, Thailand, between November 2016 and April 2019. We enrolled patients aged ≥18 years with suspected smear-negative or sputum-scarce PTB who met the following criteria:

1. Evidence of PTB based on chest imaging, with or without clinical symptoms: (a) Chest radiography, with or without chest CT, demonstrating upper lobe, lingula, right middle lobe, or superior segment lower lobe infiltrates; necrotic mediastinal lymphadenopathy with associated parenchymal changes; or bilateral miliary nodules (29). (b) Clinical features compatible with active PTB, including cough ≥2 weeks, chest pain, hemoptysis, prolonged fever, night sweats, or weight loss.
2. Microbiological criteria: Negative AFB smears from three consecutive days of self-expectorated sputum or sputum-scarce status (<1 mL of collected specimen) (5, 6, 8).

Patients were excluded if they had asthma, chronic obstructive pulmonary disease (COPD), uncontrolled hypertension, cardiac arrhythmias, allergy to β₂-agonists, prior anti-TB treatment within 2 months, pregnancy, or undiagnosed causes of chest symptoms with abnormal imaging.

This study was conducted in accordance with relevant guidelines and regulations, including the Declaration of Helsinki. The study protocol was approved by the Ethics Committee of Prince of Songkla University (IRB No. 59-205-14-1). The trial was retrospectively registered with the Thai Clinical Trials Registry (TCTR20250201002) on 1 February 2025 due to an administrative oversight at the time of study initiation. All study procedures, eligibility criteria, and outcome measures were predefined prior to participant enrollment and remained unchanged throughout the study. The study is reported in accordance with the Consolidated Standards of Reporting Trials (CONSORT) guidelines.

### Study objectives

The primary objective was to assess and compare the overall diagnostic yield of three consecutive SI procedures using nebulized racemic salbutamol vs 3% NaCl for PTB diagnosis by AFB smear or MTB culture. The secondary objective was to evaluate the cumulative and incremental yields and to compare the safety profiles of SI between the two agents. All participants provided informed consent and were randomized using computerized block randomization (block size of 4) into two groups to undergo 3 consecutive days of SI. Baseline characteristics, as well as clinical and radiological findings, were collected. SI was performed via a face mask using either nebulized racemic salbutamol solution (5 mg/5 mL) or 5 mL of 3% NaCl, delivered through a 100% oxygen compressor nebulizer at 10 L/min in a standard negative-pressure isolation room for a total of 20 min. An unblinded respiratory nurse prepared the sterile nebulizer solutions and stored them in opaque, sealed, sequentially numbered containers, while both patients and investigators remained blinded to the treatment assignment.

Following nebulization, participants were asked to cough and expectorate sputum every 5 min for up to 1 h. Spot sputum samples were collected in sterile containers and measured (15). Vital signs and induction-related AEs (19) were monitored by a single investigator (N.U.) at baseline, throughout the procedure, and every 15 min for 1 h post-nebulization. If symptomatic bronchospasm occurred—defined as wheezing with tachypnea or oxygen desaturation <95%—a rescue bronchodilator (nebulized salbutamol) was administered and SI was discontinued. Patients requiring a rescue bronchodilator prior to producing an adequate sputum sample were reassigned to the salbutamol group at the completion of the trial. Consequently, participants initially allocated to the 3% NaCl group were reassigned to the salbutamol group for the final analysis in accordance with the predefined protocol.

### Sputum processing

All sputum samples were processed within 2 h of collection. Smears were prepared and stained with auramine-rhodamine and then examined for AFB under a LED fluorescence microscope within 24 h. Positive smears were graded according to the International Union Against Tuberculosis and Lung Disease guidelines. Smears negative on fluorescence were confirmed with Ziehl-Neelsen staining, examining at least 100 fields before reporting as negative (30).

For culture, each sample was simultaneously inoculated on solid (Lowenstein-Jensen) and liquid (Middlebrook 7H9) media (4). Positive cultures were confirmed by smear microscopy. Cultures showing no growth by week 8 were considered negative. Smear and culture results were independently interpreted by two experienced laboratory technologists blinded to clinical data; discrepancies were resolved by discussion. Patients with negative AFB smears after SI underwent flexible bronchoscopy with bronchoalveolar lavage (BAL), with or without transbronchial lung biopsy, to establish a definitive diagnosis. Lung biopsy specimens were reviewed by pathologists blinded to all clinical data. Empirical anti-tuberculosis therapy was offered to patients who declined bronchoscopy.

Definitive diagnosis of PTB was based on any of the following criteria (31):

1. MTB detected in cultures of induced sputum or BAL specimens.
2. Histological examination of transbronchial biopsy showing caseating or necrotizing granulomas (32), plus clinical response to a full course of anti-TB therapy.
3. Chest imaging and/or clinical features suggestive of PTB, with clinical and radiological response to a full course of anti-TB treatment.

### Statistical analysis

Sample size was calculated using two independent proportions with 80% power. Expected diagnostic yields for 3% NaCl and salbutamol were 10.0% (13) and 22.0% (19), respectively, requiring 146 participants per group (*α* = 0.05). Allowing for 15% incomplete data, the final sample size was 168 per group. Continuous variables were analyzed using mean ± SD with Student’s *t*-test or Mann–Whitney *U* test and categorical variables with the chi-square or Fisher’s exact test. Analyses were performed in SPSS version 30.0, with *P* < 0.05 considered significant.

## RESULTS

**Fig 1:**
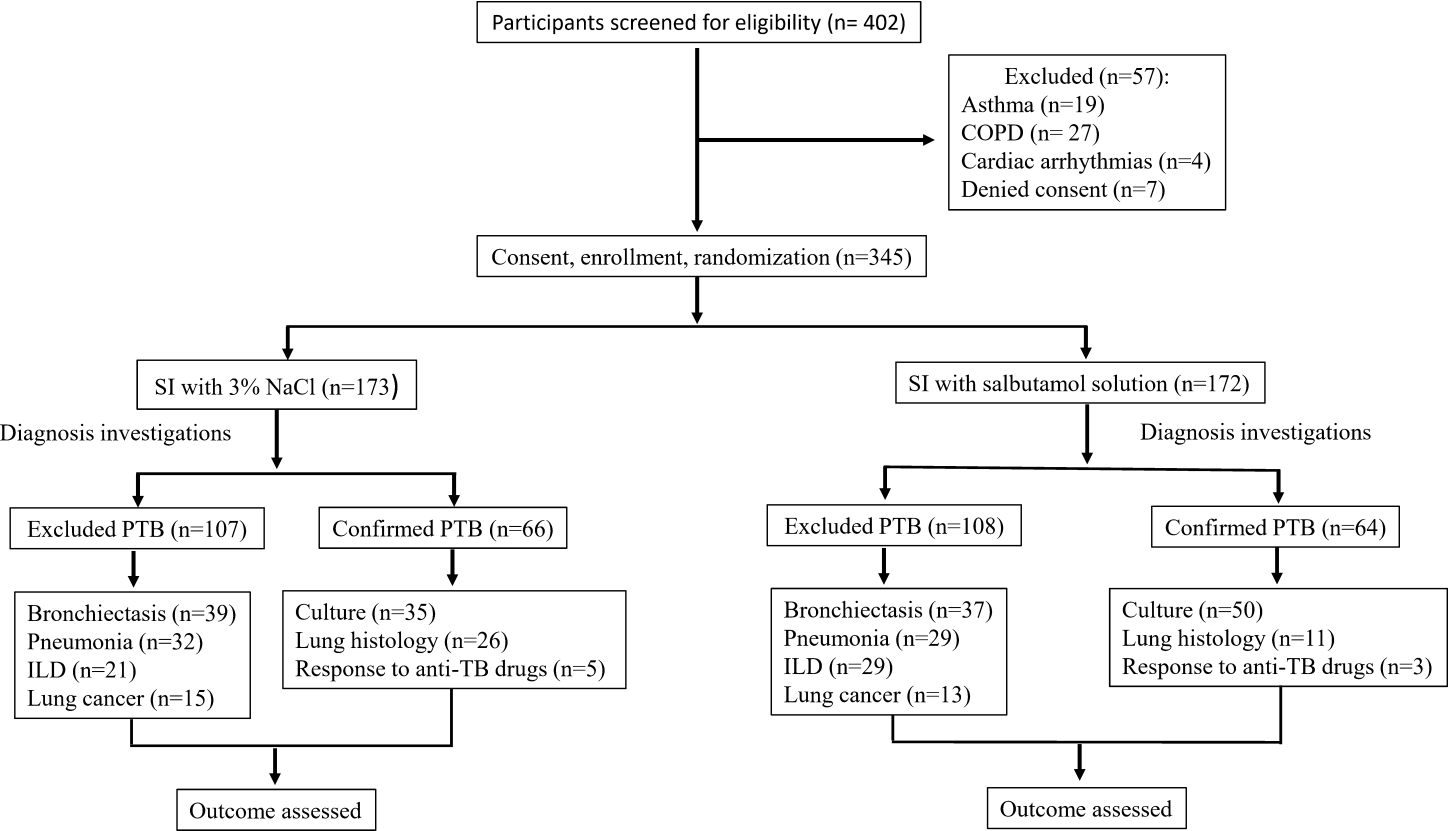
Consort flow diagram. Abbreviations: COPD, chronic obstructive pulmonary disease; SI, sputum induction; PTB, pulmonary tuberculosis; ILD, interstitial lung diseases.

Of 402 participants screened, 57 were excluded (19 asthma, 27 COPD, 4 arrhythmias, and 7 refusals), leaving 345 for randomization. Of these, 173 received 3% NaCl and 172 received nebulized salbutamol. All inductions were completed within 20 min, making the intention-to-treat and per-protocol analyses identical. Induced sputum volume was similar between groups (2.9 ± 0.2 vs 3.1 ± 0.4 mL, *P* = 0.450). After evaluation, 215 were classified as non-PTB, and 130 had confirmed PTB (66 in the 3% NaCl group and 64 in the salbutamol group) (Fig. 1). Baseline demographic and clinical characteristics did not differ significantly between groups, including age, sex, anthropometric data, smoking history, comorbidities, baseline sputum features, and chest imaging findings (Table 1).

**TABLE 1:** Baseline characteristics of patients in the nebulized 3% sodium chloride (NaCl) and racemic salbutamol solution groups^*a*^

Characteristics	3% NaCl (*n* = 173)	Salbutamol (*n* = 172)	*P*-value
Age (year), mean (SD)	48.9 (16.7)	47.6 (18.4)	0.519
Male gender, *n* (**%**)	31 (46.9)	33 (51.5)	0.097
Weight (kg), mean (SD)	47.5 (10.7)	48.9 (9.8)	0.375
Height (cm), mean (SD)	155.8 (8.7)	156.6 (9.4)	0.865
Baseline sputum characteristics			0.649
Negative AFB smear for 3 days, *n* (%)	126 (72.8)	132 (76.7)	
Sputum-scarce, *n* (%)	47 (27.2)	40 (23.3)	
Smoking history			
Never smoked, *n* (%)	71 (40.9)	78 (45.3)	0.746
Ex**-**smokers, *n* (%)	42 (24.3)	40 (23.3)	
Current smokers, *n* (%)	60 (34.8)	54 (31.4)	
**≤**20 pack**-**year	31 (18.0)	28 (16.2)	0.253
>20 pack**-**year	29 (16.8)	26 (15.2)	
Underlying diseases, *n* (%)	68 (39.3)	83 (48.2)	0.073
Diabetic mellitus, *n* (%)	23 (13.2)	20 (11.6)	
HIV, *n* (%)	13 (7.5)	10 (5.8)	
Non**-**lung cancers, *n* (%)	15 (8.7)	17 (9.9)	
Steroid use, *n* (%)	5 (2.9)	7 (4.0)	
Others^*b*^, *n* (%)	12 (7.0)	29 (16.9)	
Abnormal locations on chest radiography			0.413
Upper lobe, *n* (%)	118 (68.2)	115 (67.1)	
Middle lobe, *n* (%)	19 (10.7)	11 (6.4)	
Lower lobe, *n* (%)	10 (6.0)	14 (7.8)	
Multi**-**lobar, *n* (%)	26 (15.1)	32 (18.7)	
Abnormal patterns on chest imaging			0.625
Reticulonodular, *n* (%)	53 (30.4)	49 (28.2)	
Reticular, *n* (%)	34 (19.7)	40 (23.4)	
Patchy, *n* (%)	34 (19.7)	29 (17.1)	
Cavity, *n* (%)	29 (16.6)	32 (18.8)	
Nodular, *n* (%)	23 (13.6)	22 (12.5)	

^*a*^ 3% NaCl, 3% sodium chloride; AFB, acid-fast bacilli; HIV, human immunodeficiency virus.

^*b*^ Others: in the 3% NaCl group (*n* = 12), hypertension (4), dyslipidemia (6), and gout (2); in the salbutamol group (*n* = 29), hypertension (9), dyslipidemia (14), knee osteoarthritis (4), and hypothyroidism (2).

For the primary objective, the overall proportion of positive AFB smears from three consecutive SI procedures was comparable between the 3% NaCl and salbutamol groups (7.0% [12 cases] vs 6.4% [11 cases], *P* = 0.985). However, salbutamol yielded a significantly higher MTB culture positivity rate (15.1% vs 8.1%, *P* = 0.047). The absolute difference in culture positivity was 7.0%, corresponding to a number needed to treat (NNT) of 14. Consequently, the overall diagnostic yield based on AFB smear or MTB culture was significantly higher with salbutamol than with 3% NaCl (18.0% vs 9.2%, *P* = 0.043) (Table 2).

**TABLE 2:** Overall diagnostic yield of three consecutive sputum inductions with nebulized 3% sodium chloride (NaCl) vs nebulized racemic salbutamol solution in smear-negative or sputum-scarce pulmonary tuberculosis cases^*a*^^,^^*b*^

Characteristics	3% NaCl (*n* = 173)	Salbutamol (*n* = 172)	*P-*value
AFB smear-positive	12 (7.0%) [0.038, 0.100]	11 (6.4%) [0.034, 0.094]	0.985
MTB culture-positive	14 (8.1%) [0.044, 0.118]	26 (15.1%) [0.098, 0.204]	0.047
AFB smear- or MTB culture-positive	16 (9.2%) [0.051, 0.133]	31 (18.0%) [0.117, 0.243]	0.043

^*a*^ 3% NaCl, 3% sodium chloride; AFB, acid fast bacilli; MTB, *M. tuberculosis.*

^*b*^ The values in square brackets represent the 95% Confidence Interval (CI) for each group.

In the additional analysis, the cumulative and incremental yields of three consecutive SI for PTB diagnosis based on positive AFB smears were comparable between the two groups (*P* > 0.050) (Table 3). However, for MTB culture, the cumulative diagnostic yields from the three consecutive induced sputum samples were significantly higher in the salbutamol group than in the 3% NaCl group (8.7% vs 5.8%; 11.0% vs 6.9%; 15.1% vs 8.1% for the first, second, and third samples, respectively; all *P* < 0.050). Similarly, the incremental yields (IY) for MTB culture were significantly greater in the salbutamol group (8.7% vs 5.8%; 2.3% vs 1.2%; 4.1% vs 1.2% for the first, second, and third samples, respectively; all *P* < 0.050) (Table 3).

**TABLE 3:** Cumulative and incremental diagnostic yield of three consecutive sputum induction with nebulized 3% sodium chloride (NaCl) vs nebulized racemic salbutamol solution in smear-negative or sputum-scarce pulmonary tuberculosis cases^*a*^^,^^*b*^

	3% NaCl (*n* = 173)		Salbutamol (*n* = 172)	
	Cumulative yield	Incremental yield	Cumulative yield	Incremental yield
3.1 AFB smear-positive				
First specimen SI, *n* (%)	7 (4.0) [0.010, 0.069]	7 (4.0) [0.010, 0.069]	6 (3.5) [0.007, 0.063]	6 (3.5) [0.007, 0.063]
Second specimen SI, *n* (%)	9 (5.2) [0.023, 0.081]	2 (1.2) [0.012, 0.012]	10 (5.8) [0.023, 0.093]	4 (2.3) [0.016, 0.030]
Third specimen SI, *n* (%)	12 (7.0) [0.032, 0.108]	3 (1.8) [0.009, 0.027]	11 (6.4) [0.027, 0.101]	1 (0.6) [0.004, 0.008]
3.2 MTB culture-positive				
First specimen SI, *n* (%)	10 (5.8) [0.026, 0.090]	10 (5.8) [0.026, 0.090]	15 (8.7) [0.042, 0.132]	15 (8.7) [0.042, 0.132]
Second specimen SI, *n* (%)	12 (6.9) [0.035, 0.103]	2 (1.2) [0.009, 0.014]	19 (11.0) [0.061, 0.159]	4 (2.3) [0.016, 0.030]
Third specimen SI, *n* (%)	14 (8.1) [0.037, 0.125]	2 (1.2) [0.009, 0.044]	26 (15.1) [0.780, 0.224]	7 (4.1) [0.027, 0.073]

^*a*^ AFB, acid fast bacilli; 3% NaCl, 3% sodium chloride; SI, sputum induction; MTB, *M. tuberculosis.*

^*b*^ The values in square brackets represent the 95% Confidence Interval (CI) for each group.

Among sputum induction-related adverse events (AEs), the overall AE rate was significantly higher in the 3% NaCl group than in the salbutamol group (28.3% vs 9.8%, *P* = 0.034) (Table 4). Specifically, bronchospasm occurred only in the 3% NaCl group (13.3% vs 0.0%, *P* < 0.001), and chest tightness was also more frequent in the 3% NaCl group (8.1% vs 2.3%, *P* = 0.031), whereas cough (5.8% vs 4.7%, *P* = 0.985) and palpitation (1.6% vs 2.9%, *P* = 0.374) were comparable between groups. All 23 cases of bronchospasm in the 3% NaCl group occurred after sputum collection and resolved with 1–2 doses of nebulized salbutamol, allowing for discharge within 2–4 h. Consequently, no patients were reassigned to the salbutamol group for the analysis.

**TABLE 4:** Three consecutive sputum induction-related adverse events between nebulized 3% sodium chloride (NaCl) vs nebulized racemic salbutamol solution^*a*^^,^^*b*^

Adverse events	3% NaCl (*n* = 173)	Salbutamol (*n* = 172)	*P-*value
Overall adverse events, *n* (%)	49 (28.3) [0.221, 0.345]	17 (9.8) [0.051, 0.145]	0.034
Bronchospasm	23 (13.3) [0.089, 0.177]	0 (0.0) [0.0, 0.0]	<0.001
Cough	10 (5.8) [0.027, 0.089]	8 (4.7) [0.019, 0.075]	0.985
Chest tightness	14 (8.1) [0.045, 0.117]	4 (2.3) [0.004, 0.042]	0.031
Palpitation	2 (1.6) [0.001, 0.031]	5 (2.9) [0.012, 0.046]	0.374

^*a*^ 3% NaCl, 3% sodium chloride.

^*b*^ The values in square brackets represent the 95% Confidence Interval (CI) for each group.

## DISCUSSION

In this double-blind RCT, we evaluated the diagnostic performance of three consecutive sputum inductions (SI) using nebulized racemic salbutamol for diagnosing PTB in patients who were smear-negative or sputum-scarce. We found that nebulized salbutamol provided a significantly higher overall diagnostic yield compared with nebulized 3% hypertonic saline. This difference was primarily driven by increased culture positivity rather than smear positivity. This finding further confirms that repeated SI with nebulized salbutamol markedly increases both cumulative and incremental MTB culture yields. In addition, salbutamol was associated with substantially fewer adverse events—up to a threefold reduction—and did not induce symptomatic bronchospasm.

Delays in diagnosing tuberculosis or initiating treatment without bacteriological confirmation are linked to increased mortality (11, 33, 34) often due to inadequate sputum samples or negative AFB smears after self-expectoration (9, 34, 35). Although bronchoscopy and SI offer comparable diagnostic yields (12), bronchoscopy is invasive, less tolerated, costly, associated with complications, and often unavailable in resource-limited settings. Thus, it is not suitable as an initial diagnostic test, and SI should be performed before bronchoscopy (3).

Hypertonic saline (3%–7%) nebulization stimulates cough and draws water into the bronchial lumen, rehydrating mucus and improving clearance. SI with hypertonic saline increases PTB diagnostic yield (pooled 74%) further improved with repeated procedures (13–15). However, it can cause adverse effects, especially bronchospasm, which may require rescue bronchodilators and lead to procedure termination in up to 23% of cases. Nebulized β₂-agonists have been evaluated as safer alternative induction agents (13, 17–22).

β₂-Agonists, such as salbutamol and fenoterol, enhance mucociliary clearance by increasing secretory cells, stimulating chloride and water secretion, relaxing airway smooth muscles, and improving cough and mucus clearance (23–26). In 2015, our first RCT comparing a single SI with 5 mg nebulized salbutamol vs 3% NaCl in 147 smear-negative PTB suspects showed comparable sputum volumes and diagnostic yields (AFB 15% vs 13%, MTB culture 22% vs 17%; *P* = 0.5) but less chest tightness (5% vs 15%) and lower bronchospasm rates (0% vs 11.3%; *P* = 0.02) (19). However, only participants were blinded, and a single induction was performed. In this double-blind RCT of 130 smear-negative or sputum-scarce PTB patients, three consecutive salbutamol-induced sputum samples significantly improved overall diagnostic yield vs 3% NaCl (18.0% vs 9.2%, *P* = 0.043) and cumulative MTB culture yields (first, second, third samples: 8.7% vs 5.8%, 11.0% vs 6.9%, 15.1% vs 8.1%; all *P* < 0.05). These findings confirm the superior incremental yield of salbutamol, likely due to longer-lasting mucociliary clearance enhancement compared with hypertonic saline (25, 36–38).

It is noteworthy that the incremental yield for AFB smear positivity in the second and third salbutamol-induced specimens was not significantly higher than that of 3% NaCl. This may be because a positive smear requires a much higher bacillary load in the induced sputum than a positive culture (approximately 5,000–10,000 vs 10–100 acid-fast bacilli/mL), whereas the smear-negative PTB population we focused on represents a group with inherently low bacillary loads (7).

Directed toward the adverse effects (AEs) of the induction agents, our results showed that 3% NaCl caused substantially more overall AEs than salbutamol solution—up to a threefold increase (28.3% vs 9.8%, *P* = 0.034)—and also significantly induced symptomatic bronchospasm (13.3% vs 0.0%) (Table 4). All patients who developed bronchospasm required rescue bronchodilators, further emphasizing the limitations of 3% NaCl. These findings are consistent with our previous RCT (19) and suggest that induction using hypertonic saline may have implications for patient safety, delay hospital discharge, and increase total healthcare costs.

Besides nebulized salbutamol, in 2025, Mingchay et al. (28) conducted a participant-blind RCT comparing one to three rounds of SI using a combination regimen—nebulized bronchodilators (fenoterol 0.62 mg and ipratropium bromide 0.25 mg in 2 mL) plus 3 mL of 3% NaCl—vs 5 mL of 3% NaCl alone in 208 suspected PTB patients who had negative initial spot sputum Xpert MTB/RIF Ultra results or were sputum-scarce. The results revealed that PTB detection rates by Xpert MTB/RIF Ultra were significantly higher in the combination group than in the 3% NaCl group (30.1% vs 17.8%, *P* = 0.04). Additionally, the bronchodilator group showed a trend toward higher MTB culture positivity (31.1% vs 20.8%, *P* = 0.09) and a reduced need for bronchoscopy. Overall, AEs were mild and comparable between groups, with no reported cases of bronchospasm. These findings support the advantages of nebulized β₂-agonists and highlight the limitations of induced sputum using hypertonic saline.

**Fig 2:**
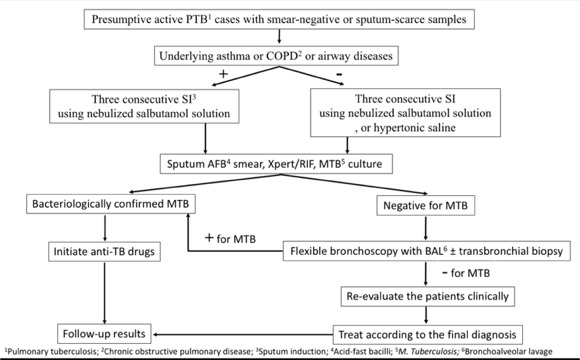
Diagnostic algorithm for patients with presumptive active PTB who are smear-negative or have sputum-scarce samples, prioritizing three consecutive SI procedures. Abbreviations: PTB, pulmonary tuberculosis; COPD, chronic obstructive pulmonary disease; SI, sputum induction; AFB, acid-fast bacilli; MTB, *M. tuberculosis;* BAL, bronchoalveolar lavage

Based on the results of previous RCTs (19, 28) and the latest evidence from our present double-blind RCT, we propose a diagnostic algorithm for patients with presumptive active PTB who are smear-negative or have sputum-scarce samples, prioritizing three consecutive SI procedures (Fig. 2).

To the best of our knowledge, this is the largest randomized, double-blind RCT that has comprehensively evaluated and compared the overall and cumulative diagnostic yields, as well as the AEs, of three consecutive induced sputum procedures using nebulized racemic salbutamol vs conventional hypertonic saline in patients with smear-negative or sputum-scarce PTB. The major strengths of our study include the following:

1. We clearly defined the criteria for smear-negative and sputum-scarce populations;
2. The details of the SI procedures, sputum processing, AEs monitoring, the definition of symptomatic bronchospasm, and the diagnostic microbiological methods and interpretations were explicitly described, ensuring optimized study design and minimizing bias;
3. We employed a single bronchodilator (salbutamol) rather than combining it with an anticholinergic bronchodilator such as ipratropium bromide because the latter lacks properties that increase sputum volume and enhance mucociliary clearance (39). Additionally, we avoided mixing salbutamol with 3% NaCl in the same solution to eliminate any potential AEs associated with hypertonic saline; and
4. We evaluated the safety profiles and determined the incremental yields of the second and third salbutamol-induced sputum specimens for MTB culture. The increased culture yield not only identified additional new index cases but also helped clarify MDR-TB cases (2 of 26 [11.5%] culture-positive cases in the salbutamol group were confirmed as MDR-TB), thereby facilitating appropriate anti-TB drug selection.

However, we also acknowledge several limitations of our study. First, we did not use the Xpert MTB/RIF assay as the confirmatory test for PTB despite its well-recognized higher sensitivity compared with AFB smear microscopy (40). This decision was driven by concerns regarding its cost and limited availability in many healthcare institutions in Thailand; consequently, this may have reduced the overall diagnostic yield in our study. Second, this study included a small subset of patients (*n* = 8) diagnosed with PTB based on clinical and radiological response to anti-tuberculosis treatment rather than microbiological confirmation (31), which may introduce potential classification bias. Although a sensitivity analysis excluding these cases was planned to assess robustness, it could not be performed due to the unavailability of the complete individual-level data set following data migration. Nevertheless, given that these cases represent a minimal proportion (6.1%) of the total cohort, their exclusion is unlikely to meaningfully alter the primary findings or the comparative diagnostic yield between the two groups. Third, we did not compare the diagnostic yield of nebulized therapy with other delivery methods, such as a metered-dose inhaler (MDI), as there is no clear or consistent evidence that one method is markedly superior in terms of mucociliary clearance. Fourth, this study did not compare the diagnostic yields of three consecutive SI sessions using nebulized salbutamol versus fenoterol. Lastly, we did not perform RCT in specific patient groups such as those with asthma or COPD due to ethical concerns, as these patients are at increased risk of severe bronchospasm when undergoing SI with hypertonic saline. Therefore, further RCTs assessing and comparing the diagnostic yields of three consecutive SI sessions using different delivery methods and types of β₂-agonists, such as fenoterol, as well as incorporating the Xpert MTB/RIF assay as a confirmatory test for MTB, are needed to address these limitations.

### Conclusions

In patients with smear-negative or sputum-scarce PTB, three consecutive sputum inductions using nebulized salbutamol provided higher culture positivity, resulting in a greater overall diagnostic yield compared with conventional 3% hypertonic saline. Repeated SI with nebulized salbutamol also increases both cumulative and incremental culture yields. In addition, repeated induced sputum collection with salbutamol was safe and noninvasive method. Therefore, nebulized salbutamol should be considered a preferred induction agent for SI procedures, particularly in high-risk patients such as those with airway diseases.

## References

[1] World Health Organization (WHO). 2024. Global Tuberculosis Report 2024. Geneva, WHO

[2] Melsew YA, Doan TN, Gambhir M, Cheng AC, McBryde E, Trauer JM. 2018. Risk factors for infectiousness of patients with tuberculosis: a systematic review and meta-analysis. Epidemiol Infect 146:345–353. doi:10.1017/S0950268817003041 29338805 PMC9134570

[3] Lewinsohn DM, Leonard MK, LoBue PA, Cohn DL, Daley CL, Desmond E, Keane J, Lewinsohn DA, Loeffler AM, Mazurek GH, O’Brien RJ, Pai M, Richeldi L, Salfinger M, Shinnick TM, Sterling TR, Warshauer DM, Woods GL. 2017. Official American thoracic society/infectious diseases society of america/centers for disease control and prevention clinical practice guidelines: diagnosis of tuberculosis in adults and children. Clin Infect Dis 64:e1–e33. doi:10.1093/cid/ciw694 27932390

[4] Gill CM, Dolan L, Piggott LM, McLaughlin AM. 2022. New developments in tuberculosis diagnosis and treatment. Breathe 18:210149. doi:10.1183/20734735.0149-2021 35284018 PMC8908854

[5] Campos LC, Rocha MVV, Willers DMC, Silva DR. 2016. Characteristics of patients with smear-negative pulmonary tuberculosis (TB) in a region with high TB and HIV prevalence. PLoS One 11:e0147933. doi:10.1371/journal.pone.0147933 26808299 PMC4725950

[6] World Health Organization. 2007. Improving the diagnosis and treatment of smear-negative pulmonary and extrapulmonary tuberculosis among adults and adolescents. Recommendations for HIV prevalent and resource-constrained settings. Available from: www.who.int

[7] Colebunders R, Bastian I. 2020. A review of the diagnosis and treatment of smear-negative pulmonary tuberculosis. Int J Tuberc Lung Dis 4:97–107. 10694086

[8] Zuberi FF, Hussain S, Hameed S, Zuberi BF. 2019. Role of bronchial washing gene xpert in sputum-scarce cases of suspected pulmonary tuberculosis. Pak J Med Sci 35:211–214. doi:10.12669/pjms.35.1.107 30881425 PMC6408649

[9] Zhang Y, Zhan B, Hao X, Wang W, Zhang X, Fang C, Wang M. 2023. Factors associated with diagnostic delay of pulmonary tuberculosis among children and adolescents in Quzhou, China: results from the surveillance data 2011–2021. BMC Infect Dis 23:541. doi:10.1186/s12879-023-08516-1 37596514 PMC10439644

[10] Asadi L, Croxen M, Heffernan C, Dhillon M, Paulsen C, Egedahl ML, Tyrrell G, Doroshenko A, Long R. 2022. How much do smear-negative patients really contribute to tuberculosis transmissions? Re-examining an old question with new tools. EClinicalMedicine 43:101250. doi:10.1016/j.eclinm.2021.101250 35036885 PMC8743225

[11] Obeagu EI. 2023. Tuberculosis diagnostic and treatment delays among patients in Uganda. Health Sci Rep 6:e1700. doi:10.1002/hsr2.1700 38028687 PMC10651951

[12] Luo W, Lin Y, Li Z, Wang W, Shi Y. 2020. Comparison of sputum induction and bronchoscopy in diagnosis of sputum smear-negative pulmonary tuberculosis: a systemic review and meta-analysis. BMC Pulm Med 20:146. doi:10.1186/s12890-020-01192-w 32450826 PMC7249394

[13] Hepple P, Ford N, McNerney R. 2012. Microscopy compared to culture for the diagnosis of tuberculosis in induced sputum samples: a systematic review. Int J Tuberc Lung Dis 16:579–588. doi:10.5588/ijtld.11.0617 22410498

[14] Schoch OD, Rieder P, Tueller C, Altpeter E, Zellweger J-P, Rieder HL, Krause M, Thurnheer R. 2007. Diagnostic yield of sputum, induced sputum, and bronchoscopy after radiologic tuberculosis screening. Am J Respir Crit Care Med 175:80–86. doi:10.1164/rccm.200608-1092OC 17053204

[15] Chanez P, Holz O, Ind PW, Djukanović R, Maestrelli P, Sterk PJ, Leader of the Working Group: P.L. Paggiaro1, Members of the Working Group: 2002. Sputum induction. Eur Respir J 20:3s–8s. doi:10.1183/09031936.02.00000302 12361361

[16] Gonzalez-Angulo Y, Wiysonge CS, Geldenhuys H, Hanekom W, Mahomed H, Hussey G, Hatherill M. 2012. Sputum induction for the diagnosis of pulmonary tuberculosis: a systematic review and meta-analysis. Eur J Clin Microbiol Infect Dis 31:1619–1630. doi:10.1007/s10096-011-1485-6 22095153

[17] Zahrani KA, Jahdali HA, Poirier L, Rene P, Menzies D. 2001. Yield of smear, culture and amplification tests from repeated sputum induction for the diagnosis of pulmonary tuberculosis. Int J Tuberc Lung Dis 5:855–860. 11573898

[18] de la Fuente PT, Romagnoli M, Godard P, Bousquet J, Chanez P. 1998. Safety of inducing sputum in patients with asthma of varying severity. Am J Respir Crit Care Med 157:1127–1130. doi:10.1164/ajrccm.157.4.9610008 9563729

[19] Keeratichananont W, Nilmoje T, Keeratichananont S, Rittatorn J. 2015. Diagnostic yield and safety of sputum induction with nebulized racemic salbutamol versus hypertonic saline in smear-negative pulmonary tuberculosis. Ther Adv Respir Dis 9:217–223. doi:10.1177/1753465815594529 26206666

[20] Geldenhuys HD, Kleynhans W, Buckerfield N, Tameris M, Gonzalez Y, Mahomed H, Hussey G, Hanekom W, Hatherill M. 2012. Safety and tolerability of sputum induction in adolescents and adults with suspected pulmonary tuberculosis. Eur J Clin Microbiol Infect Dis 31:529–537. doi:10.1007/s10096-011-1344-5 21796347

[21] Makris D, Tzanakis N, Moschandreas J, Siafakas NM. 2006. Dyspnea assessment and adverse events during sputum induction in COPD. BMC Pulm Med 6:17. doi:10.1186/1471-2466-6-17 16808839 PMC1524986

[22] T. Ricci C de A, F. Lasmar LM de LB, Pitrez PM, F. Mascarenhas R, Camargos PAM. 2015. Sputum induction in children and adolescents with problematic severe asthma: success rate, safety and tolerability. TOALLJ 8:7–13. doi:10.2174/1874838401508010007

[23] Bennett WD. 2002. Effect of beta-adrenergic agonists on mucociliary clearance. J Allergy Clin Immunol 110:S291–7. doi:10.1067/mai.2002.129704 12464938

[24] Yazdani A, Kiran A, Murthy KJ. 2002. Sputum induction by oral salbutamol. Ind J Tub 49:221–223.

[25] Hasani A, Toms N, Agnew JE, Lloyd J, Dilworth JP. 2005. Mucociliary clearance in COPD can be increased by both a D2/beta2 and a standard beta2 agonists. Respir Med 99:145–151. doi:10.1016/j.rmed.2004.07.004 15715181

[26] Weich DJ, Viljoen H, Sweetlove MA, Pretorius PH, van Rensburg AJ, Lötter MG, Minnaar PC, Otto AC. 1988. Investigation into the effect of Fenoterol on mucociliary clearance in patients with chronic bronchitis. Eur J Nucl Med 14:533–537. doi:10.1007/BF00286771 3208783

[27] Ansari MSS, Hidayath M, Kawoosa W, Ghouse A. 2013. A comparative study of sputum induction in suspected pulmonary tuberculosis. Biol Med 5:83–90.

[28] Mingchay P, Kawkitinarong K, Torvorapanit P, Ohata PJ, Suwanpimolkul G. 2026. Effectiveness of nebulized bronchodilator-enhanced sputum induction in Thai patients with presumed pulmonary tuberculosis: a randomized controlled trial (NeB-TB Trial. Clin Infect Dis 82:427–435. doi:10.1093/cid/ciaf580 41125243

[29] Bhatia H, Kaur R, Kaur I, Garg K. 2024. Imaging spectrum in pulmonary tuberculosis. Indographics 03:033–044. doi:10.1055/s-0044-1788611

[30] Gizaw N, Abera A, Sisay S, Desta K, Kreibich S, Gerwing-Adima L, Gebre-Selassie S. 2020. The yield of auramine O staining using led microscopy with bleach treated sputum samples for detection of pulmonary tuberculosis at St. Peter tuberculosis specialized hospital, Addis Ababa, Ethiopia. J Clin Tuberc Other Mycobact Dis 18:100140. doi:10.1016/j.jctube.2019.100140 31909226 PMC6939099

[31] World Health Organization. 2013. Definitions and reporting framework for tuberculosis – 2013 revision: updated December 2014 and January 2020. World Health Organization. Available from: https://iris.who.int/handle/10665/79199

[32] Desai HM, Vaideeswar P, Gaikwad M, Amonkar GP. 2022. Pathology of pulmonary tuberculosis: has the tiger changed it’s stripes? Autops Case Rep 12:e2021370. doi:10.4322/acr.2021.370 35496733 PMC9037862

[33] Bea S, Lee H, Choi WS, Huh K, Jung J, Shin JY. 2023. Risk of mortality and clinical outcomes associated with healthcare delay among patients with tuberculosis. J Infect Public Health 16:1313–1321. doi:10.1016/j.jiph.2023.05.038 37339564

[34] Freitag B, Sultanli A, Grilli M, Weber SF, Gaeddert M, Abdullahi OA, Denkinger CM, Gupta-Wright A. 2025. Clinically diagnosed tuberculosis and mortality in high burden settings: a systematic review and meta-analysis. EClinicalMedicine 84:103251. doi:10.1016/j.eclinm.2025.103251 40496884 PMC12146525

[35] Teo AKJ, Singh SR, Prem K, Hsu LY, Yi S. 2021. Duration and determinants of delayed tuberculosis diagnosis and treatment in high-burden countries: a mixed-methods systematic review and meta-analysis. Respir Res 22:251. doi:10.1186/s12931-021-01841-6 34556113 PMC8459488

[36] Matthys H, Daikeler G, Krauss B, Vastag E. 1987. Action of tulobuterol and fenoterol on the mucociliary clearance. Respiration 51:105–112. doi:10.1159/000195176 2884705

[37] Weiss T, Dorow P, Felix R. 1981. Effects of a beta adrenergic drug and a secretolytic agent on regional mucociliary clearance in patients with COLD. Chest 80:881–885. doi:10.1378/chest.80.6.881 7030660

[38] Bennett WD, Burbank A, Almond M, Wu J, Ceppe A, Hernandez M, Boucher RC, Peden DB. 2021. Acute and durable effect of inhaled hypertonic saline on mucociliary clearance in adult asthma. ERJ Open Res 7:00062–02021. doi:10.1183/23120541.00062-2021 34109248 PMC8184161

[39] Taylor RG, Pavia D, Agnew JE, Lopez-Vidriero MT, Newman SP, Lennard-Jones T, Clarke SW. 1986. Effect of four weeks’ high dose ipratropium bromide treatment on lung mucociliary clearance. Thorax 41:295–300. doi:10.1136/thx.41.4.295 2943050 PMC460313

[40] Rasool G, Khan AM, Mohy-Ud-Din R, Riaz M. 2019. Detection of *Mycobacterium tuberculosis* in AFB smear-negative sputum specimens through MTB culture and GeneXpert MTB/RIF assay. Int J Immunopathol Pharmacol 33:2058738419827174. doi:10.1177/2058738419827174 30791749 PMC6360468

